# Design of the Park-in-Shape study: a phase II double blind randomized controlled trial evaluating the effects of exercise on motor and non-motor symptoms in Parkinson’s disease

**DOI:** 10.1186/s12883-015-0312-6

**Published:** 2015-04-16

**Authors:** Nicolien M van der Kolk, Sebastiaan Overeem, Nienke M de Vries, Roy PC Kessels, Rogier Donders, Marc Brouwer, Daniela Berg, Bart Post, Bas R Bloem

**Affiliations:** 1 Department of Neurology, Donders Institute for Brain, Cognition, and Behavior, Radboud University Medical Center Nijmegen, PO Box 9101, 6500 HB Nijmegen, The Netherlands; 2 Department of Medical Psychology, Donders Institute for Brain, Cognition and Behaviour, Radboud University Medical Center, Nijmegen, the Netherlands; 3 Department of Health Evidence, Radboud University Medical Center Nijmegen, Nijmegen, The Netherlands; 4 Department of Cardiology, Radboudumc, Nijmegen, the Netherlands; 5 Department of Neurodegeneration, Center for Neurology and Hertie-Institute for Clinical Brain Research, Tübingen, Germany

**Keywords:** Parkinson disease, Physical activity, Exergaming, RCT

## Abstract

**Background:**

Parkinson’s disease (PD) is a neurodegenerative disorder with a wide range of motor and non-motor symptoms. Despite optimal medical management, PD still results in a high disability rate and secondary complications and many patients lead a sedentary lifestyle, which in turn is also associated with a higher co-morbidity and mortality. Exercise has been explored as a strategy to reduce secondary complications and results suggests that it not only provides general health benefits, but may also provide symptomatic relief. If this holds true exercise would be a very attractive addition to the therapeutic arsenal in PD. The supportive evidence remains incomplete. Here, we describe the design of the Park-in-Shape study, which primarily aims to evaluate whether aerobic exercise affords clinically relevant improvements in motor symptoms in sedentary PD patients. A specific new element is the introduction of gaming to optimize compliance to the exercise intervention.

**Methods/Design:**

The Park-in-Shape study is a randomized controlled, assessor- and patient-blinded single center study. Two parallel groups will include a total of 130 patients, receiving either aerobic exercise on a home trainer equipped with gaming elements (“exergaming”), or a non-aerobic intervention (stretching, flexibility and relaxation exercises). Both groups are supported by a specifically designed motivational app that uses gaming elements to stimulate patients to exercise and rewards them after having completed the exercise. Both interventions are delivered at home at least 3 times a week for 30–45 minutes during 6 months. Eligible patients are community-dwelling, sedentary patients diagnosed with mild-moderate PD. The primary outcome is the MDS-UPDRS motor score (tested in the *off state*) after 6 months. Secondary outcomes include various motor and non-motor symptoms, quality of life, physical fitness, and adherence.

**Discussion:**

This Park-in-Shape study is anticipated to answer the question whether high intensity aerobic exercise combined with gaming elements (“exergaming”) provides symptomatic relief in PD. Strong elements include the double-blinded randomized controlled trial design, the MDS-UPDRS as valid primary outcome, the large sample size and unique combination of home-based pure aerobic exercise combined with gaming elements and motivational aspects.

**Trial registration:**

Dutch trial register NTR4743

## Background

Despite optimal medical management, Parkinson’s disease (PD) remains a disabling and costly neurodegenerative disease in current clinical practice. The disease-related motor and non-motor symptoms largely determine the patients’ disability and therefore their quality of life. In addition many PD patients lead a sedentary lifestyle due to these symptoms, resulting in a higher co-morbidity and mortality. Additional strategies that reduce these symptoms as well as their secondary complications are needed to expand the patients’ functional ability, optimize independence and improve quality of life. Over the last decades exercise has been explored as such a strategy and has shown promising results, with positive effects on generic health benefits as well as on disease-related symptoms [1-3]. Studies in animal models of PD have demonstrated that high-intensity exercise can provide symptomatic relief of parkinsonian signs [4]. Similar results were seen in small treadmill studies in PD patients, showing improvement in gait parameters and enhanced physical fitness [5-7]. Perhaps even more fascinating are the observed changes in the “parkinsonian” rodent striatum after aerobic exercise, including: (a) increased angiogenesis [8], (b) decreased expression of brain damage markers [9], (c) decreased expression of the dopamine active transporter, (d) increased D2 receptor mRNA levels and even (e) reversal of dopaminergic cell loss [4,10,11]. Two recent small cohort studies in PD patients show similar results following high-intensive aerobic exercise. Specifically, postsynaptic D2 receptor binding potential on PET imaging increased [12] and cortical hyperexcitability (which is characteristic for the parkinsonian state), as measured with transcranial magnetic stimulation, reduced [13]. This suggests that intensive aerobic exercise may promote adaptive plasticity of the dopaminergic system, raising hopes that exercise might change the course of PD. If this holds true, exercise would be a very attractive addition to the therapeutic arsenal in PD.

Based on these promising results, (aerobic) exercise is already widely advocated as symptomatic treatment in current practice. However, the strength of the available evidence remains limited, due to small sample sizes and the uncontrolled or non-randomized nature of previous research. In addition, heterogeneous interventions (type, intensity and duration) and outcomes were used, making a meta-analysis or systematic review challenging [2,3,14,15]. Finally, treatment compliance is major challenge in any exercise study, particularly if the exercise should be maintained for long periods (up to many years) to achieve full benefits.

Here, we describe the design of the Park-in-Shape study, a controlled, assessor and patient-blinded single centre study that addresses some of the aforementioned shortcomings. A specific new element is the introduction of gaming to optimize compliance to the exercise intervention, in three different ways: to motivate patients to *begin* the exercise; to stimulate patients *during* the actual exercise; and to reward patients *after* having completed the exercise.

### Objectives

The primary objective of the Park-in-Shape study is to evaluate whether aerobic exercise can lead to clinically relevant improvements in motor functioning in sedentary PD patients. The secondary objectives are to evaluate whether aerobic exercise results in improvements in other clinically relevant symptoms, physical fitness and quality of life.

## Methods/Design

### Ethical approval and trial registration

The study is executed in compliance with the Helsinki Declaration. The study protocol, patient information and informed consent forms have been approved by the local ethics committee (CMO Arnhem-Nijmegen; NL47747.091.14.). Informed consent is signed at the beginning of the in-person assessments, after the patient is fully informed about the procedures. The Park-in-Shape trial is registered in the Dutch trial registry (www.trialregister.nl registration number NTR4743).

### Study design

**Figure 1:**
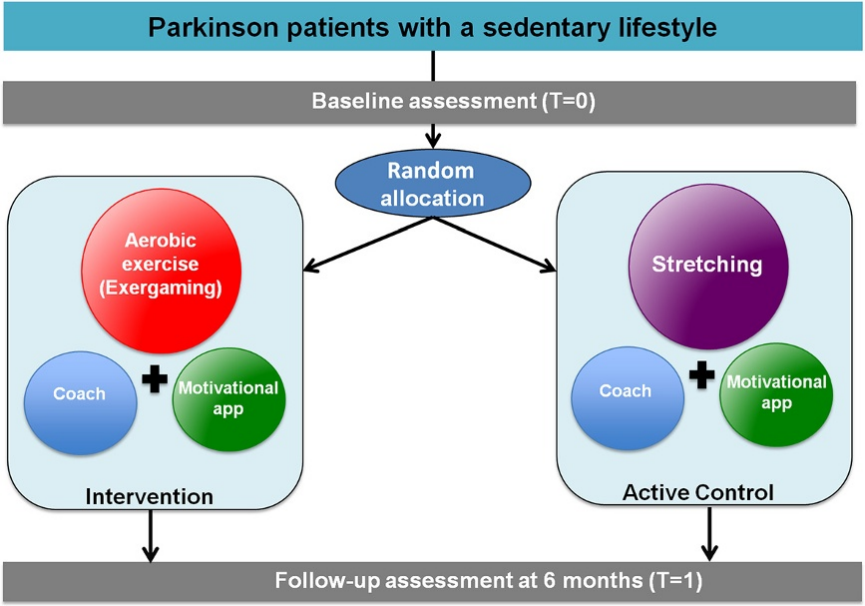
Study designPark-in-shape.

The Park-in-Shape study is a randomized controlled, double-blinded study performed in a single center (Figure 1). Patients are randomly assigned to the intervention (aerobic exercise) or the active control group (stretching), but are unaware of the allocation possibilities. The assessors performing the baseline and follow-up assessments (directly after the intervention period of 6 months) are also blinded for allocation. Motivational aspects are applied in both groups to increase compliance (see below for further details).

### Study population

In- and exclusion criteria are shown in Table 1. Included participants are community-dwelling patients with a sedentary lifestyle. All patients who performed none or insufficient aerobic physical activity according to the current recommendations for of the American College of Sports Medicine (ACSM) for older adults [16] (i.e. vigorous exercise performed < 3 times a week, 20 minutes per session or moderate exercise performed <5 times a week, 30 minutes per session) are considered to have a sedentary lifestyle and are eligible. Furthermore, eligible patients are aged between 30 and 75, have idiopathic PD diagnosed by a neurologist (using the UK Brain Bank Criteria), with Hoehn and Yahr stages I-II (tested in the *off* state) [17,18]. Both medicated and unmedicated patients are eligible. Medicated patients can only be included if they receive a stable dopaminergic medication dose for at least one month prior to inclusion in the study (levodopa and/or a dopamine agonist are allowed). Unmedicated patients are eligible if they are deemed unlikely to start treatment within the next month, as judged by their treating neurologist.

**Table 1:** **In- and exclusion criteria**

Inclusion criteria	Exclusion criteria
Idiopathic Parkinson’s disease diagnosed by a neurologist	Medication:
	• Beta-blockers and/or anti-psychotics
Hoehn & Yahr stage ≤2 (tested in *off state*)	Comorbidity:
	• Neurologic or orthopedic co-morbidities that make it impossible to cycle or perform stretching exercises (safely)
	• Contra-indications for aerobic exercise including diagnosed cardiac diseases, diagnosed but poorly controlled diabetes
	• mellitus or pulmonary diseases.
	• Psychiatric diseases: major depressive disorder, severe or moderate depressive episode or any form of psychosis), diagnosed by a psychiatrist in the last year.
	• Dementia: MMSE <24
Age 30–75 years	Inability to fill out questionnaires or perform a computer task (i.e. due to poor vision, inability to read Dutch (illiteracy or foreign language))
Sedentary lifestyle [16]	Facilities:
	• No internet at home
Parkinson medication:	Availability/compliance:
• Stable dopaminergic medication dose (both levodopa and/or a dopamine agonist are allowed) for at least one month before the study	• Unavailable for more than 10% (approximately 2.5 weeks) of the 6 months
• No treatment and deemed unlikely to start treatment within the next month by their treating neurologist	

### Recruitment and setting

Participants will be recruited primarily through the neurology department at the Radboud university medical center (Radboudumc), Nijmegen, the Netherlands. Additional centers in the vicinity of Nijmegen will be invited to assist in the recruitment. The inclusion period will last 2.5 years. Eligibility is established through telephone screening and in-person assessments. During the telephone screening a trained research nurse interviews the patients regarding their physical activity (LAPAQ outdoor and norms of the ACSM [16]), comorbidity, medication use, ability to complete questionnaires or perform computer tasks, facilities at home and availability. If there is no reason for exclusion at this stage, participants are invited to the study site for in-person assessments. In-person assessments take place in the Radboudumc and include: (a) screening of cognitive function and disease stage (Hoehn & Yahr) without dopaminergic medication by a trained research nurse, (b) medical screening by a cardiologist to rule out any medical conditions that preclude endurance exercise; and (c) a maximal-graded exercise test to determine the presence of serious arrhythmias, evidence of ischemia or increases of diastolic blood pressure above 115 mmHg or increases of systolic blood pressure above 235 mmHg. Abnormalities observed during the graded exercise test require follow-up by a cardiologist to determine whether high-intensity exercise can be performed safely. Informed consent is signed at the beginning of the in-person assessments. If no exclusion criteria are met during the in-person assessments, patients are included and baseline measurements are performed.

### Randomization and blinding

Within two weeks after informed consent is signed, participants will be randomly assigned to one of the two groups using a web-based system designed by an independent statistician. The allocation ratio is 1:1 and the procedure entails permuted blocks of varying sizes (unknown to the study personnel). Minimization is performed for gender and medication status (treated/untreated). The patients, all study personnel involved in the screening and assessments and the researcher who will analyze the data will be blinded for the allocation. Randomization is performed by a member of the research team who is not involved in the assessments or the data analysis. Patients will be informed of their allocation after randomization by the coach (see below) but are unaware of the details of the two allocation possibilities. Blinding of patients will be checked after the patient has finished his/her participation by asking them whether they felt their prescribed program was effective.

### Measurements

Assessments will be performed at baseline and after 6 months. All measurements are only performed in *off* state, except for the maximal graded exercise test which is performed in the *on* state and in the UPDRS III, which is performed in both the *off* state (primary outcome) and in the *on* state (secondary outcome). The maximal-graded exercise test is performed in *on* state for two reasons: 1) to minimize any PD related motor limitations, allowing for a maximal cardiovascular performance, and 2) because patients are also instructed to train during their participation in the study in their optimal therapeutic state (*on* state) and the training intensity will be based on the maximal heart rate determined in the maximal graded exercise test. *Off* state is determined as >12 hours after the last Parkinson medication intake (and if applicable: deep brain stimulation switched off during the measurements). Therefore all patients are asked to skip the morning dose of their Parkinson medication at the day of the assessment. All assessments will start at the same time of the day and will be performed in the same sequence, minimizing the difference in time spent in the *off* state between participants. At the end of the assessment, the two aforementioned tests are performed in the *on* state. *On* state is determined by 1 hour after taking a supra-threshold dose of their morning medication (125% of their morning levodopa equivalent dose (LED) for levodopa [19,20]). Half an hour before their lunch, patients are instructed to take this supra-threshold dose to ensure that they reach an *on* state that is equivalent to their everyday functioning, and that persists throughout the *on* state assessments. All tests and questionnaires used are described in Table 2 and are detailed here below.

**Table 2:** **Study schedule and assessments**

	t = wk 0	t = wk 1-2	t = wk 2-3	t = wk 26-27	t = wk 27-28
Activity/Assessment	Pre-study screening (phone)	Study visit 1 (Screening/Baseline)	@ home	Study visit 2 (post-intervention)	@ home
**Inclusion/Exclusion criteria**					
Cardiovascular risk factors					
Rose questionnaire	x				
Physical exam & ECG		x		x	
MMSE		x			
LAPAQ outdoor	x				
H&Y stage		x		x	
**Primary outcome**					
**Motor symptoms**					
MDS-UPDRS III *off*		x		x	
**Secondary outcomes**					
**Motor symptoms**					
MDS-UPDRS III *on*		x		x	
MDS-UPDRS IV		x		x	
TUG		x		x	
Pegboard		x		x	
Finger tap test		x		x	
Mini-BESTest		x		x	
Falls & Near-falls		x		x	
**Non-motor symptoms**					
TAP flexibility		x		x	
TMT		x		x	
MoCa		x		x	
SCOPA-sleep			x		x
HADS			x		x
SCOPA-AUT			x		x
FSS			x		x
**Quality of life**					
PDQ-39			x		x
**Physical fitness**					
VO2max		x		x	
6MWT		x		x	
**Adherence**					
Bike computer/motivational app data		Data is automatically collected after each workout on a secured server. After 6 months the data is archived and locked.			

ECG = electrocardiogram. MMSE = Mini Mental State Examination, LAPAQ = Longitudinal aging study Amsterdam Physical Activity Questionnaire. H&Y = Hoehn & Yahr, MDS-UPDRS III/IV = Movement Disorders Society-sponsored revision of the Unified Parkinson’s Disease Rating Scale, motor section (III) and motor complications section (IV). TUG = Timed Up and Go test. Mini-BESTets = Mini-Balance Evaluation System Test. TAP = Test of Attentional Performance. TMT = Trial Making Test. MoCa = Montreal Cognitive Assessment. SCOPA-sleep = SCales for Outcomes in PArkinson’s disease, sleep section. SCOPA-aut = SCales for Outcomes in PArkinson’s disease, autonomic function section. FSS = Fatigue Severity Scale. PDQ-39 = 39-item Parkinson Disease Questionnaire VO2max = maximal amount of oxygen inhaled during maximal graded test. 6MWT = 6 Minute Walk Test.

#### General variables

The following data are collected at baseline: age at baseline, gender, education, age at onset and disease duration. Details on Parkinson medication (i.e., drug name, dose, frequency, levodopa equivalence) will be collected during both visits using a customized form.

#### Primary outcome measure

The primary outcome will be the score on the motor section of the Movement Disorders Society-sponsored revision of the Unified Parkinson’s Disease Rating Scale (MDS-UPDRS) [18] after 6 months measured in the *off* state. The UDPRS is the most widely used, well-validated, clinical rating scale for PD and has been shown to be sensitive to change in clinical status [21] and data on clinical important differences are available [22,23]. Based on these data we considered a change of minimal 3.5 clinically important. Clinical progression appears to be faster and UPDRS motor score increases steeper in early stages of PD [24-26], underlining the need for treatments that can reduce the UPDRS motor score in these stages and slow down the disability rate. As intra-rater variability is lower compared to inter-rater variability [27] the same rater will perform the MDS-UDPRS-III assessments at baseline and follow up.

#### Secondary outcome measures

##### Additional motor symptoms

To test the impact of the intervention on motor symptoms during everyday life, the MDS-UPDRS III is also assessed in the *on* state. Balance deficits will be analyzed with the short version of the Balance Evaluation System Test (Mini-BESTest). The Mini-BEST test is a clinical tool that is highly correlated with the validated and generally accepted Berg Balance Scale, but is more suitable for mild PD as it has no ceiling effect [28]. Balance impairment is defined as a score of ≥21 on the Mini-BESTest [29]. The Timed Up and Go test (TUG) will be used to test mobility. The TUG measures the ability of patients to perform sequential locomotor tasks that incorporate walking and turning. It has been validated in PD and is sensitive to changes in mobility in PD patients [30]. The Dexterity device of the Objective Parkinson’s Disease Measurement system will be used to quantify bradykinesia with a digitography (keyboard) test, and complex motor functioning is tested with a pegboard test.

Motor complications and (near) falls will be assessed by questionnaires. Falls and near-falls will be registered retrospectively over a period of 6 months with an interview-based questionnaire that is used in clinical practice by physiotherapist specialized in PD [31]. Even patients with early disease may already experience fluctuations in their response to levodopa [32]. Therefore, motor complications will be evaluated as well, using the interview-based MDS-UPDRS IV questionnaire.

##### Non-motor symptoms

Several studies report beneficial effects of aerobic exercise on cognitive function in healthy elderly [33]. During the in-person assessments several cognitive tests will be performed. The Montreal Cognitive Assessment scale (MoCa) is used for a quick screening of global cognitive function. It has been widely accepted for use in PD populations [34] by assessing multiple domains of cognitive function including memory, language, complex visuospatial processing, and executive function. The MoCa is suggested to be more sensitive to cognitive impairment in PD and to temporal changes compared with the MMSE [35]. One of the earliest cognitive deficits in PD is executive function (set-shifting in particular) [36]. We will perform two tests to assess set-shifting in *off* state: the flexibility subset of the Test of Attentional Performance (TAP 3.2) and the Trail Making Test (TMT) A and B. In the flexibility subset of the TAP test two verbal stimuli (letter-number) are simultaneously presented on a computer screen and the patient has to press the button on the side of the target stimulus. The target stimulus will either be fixed (requiring a response to the side of letters or numbers only) or alternating (requiring a response that alternates between letters and numbers). A similar non-computerized version of this task has been used before in PD and shown to be highly specific for task-set shifting in PD patients in *off* state [37]. The TMT is one of the most frequently used paper and pencil test in clinical practice and consists of two parts: one assessing psychomotor speed (A) and one assessing mental flexibility (B) [38]. Also in PD the TMT is able to indicate deficits in set shifting [39]. For all three cognitive tests parallel versions will be used at follow up, to minimize learning effects.

Several studies in non-PD populations indicated beneficial effects of aerobic exercise on mood [40,41], sleep [42], constipation [43] and fatigue [44]; all of which are part of the non-motor symptoms that PD patients may experience. We will use self-rating questionnaires that are completed at home to assess these non-motor symptoms. Mood will be assessed with the Hamilton Anxiety and Depression Scale (HADS). This is a 14-item scale assessing both depression and anxiety and has been validated in PD patients [45]. The section on sleep of the Scales for Outcomes in Parkinson’s disease (SCOPA-Sleep) will be used to measure sleep disturbances. This a short practical self-rating scale designed to evaluate sleep quality and daytime sleepiness [46]. Autonomic symptoms can be assessed with the SCOPA-autonomic (SCOPA-AUT) [47]. For the purpose of this trial only the gastro-intestinal section of the SCOPA-AUT will be used to assess constipation. Fatigue will be addressed by the Fatigue Severity Scale (FSS), which has been recommended for screening and severity rating in PD by the movement disorders society [48].

##### Quality of life

Quality of life will be assessed with the 39-item Parkinson Disease Questionnaire (PDQ-39), a PD specific self-administered questionnaire [49]. Participants are asked to fill out this questionnaire at home.

##### Physical fitness

Physical fitness is measured using the 6-minute walk test and the maximal aerobic power tested by indirect calorimetry (VO2max) during the maximal graded exercise test. The 6-minute walk test is widely used to assess functional exercise capacity and normative values for PD patients exist [50]. The VO2max is the gold standard of cardiovascular fitness and is a reliable and repeatable measure in subjects with mild to moderate PD [51].

##### Adherence

Adherence will be determined based on the number of dropouts and the training frequency in both groups, as well as the total time exercised within the prescribed heart rate zone in the intervention group.

### Interventions

The total duration of the intervention will be 6 months, regardless of group allocation. All patients will be supported by the same coach during their intervention.

#### Aerobic exercise (intervention group)

The intervention is multifaceted and consists of a unique combination of home-based aerobic exercise, enhanced with virtual reality software and real-life videos, the use of a motivational app and coaching with tele-monitoring from a distance. Aerobic exercise, performed on a stationary home trainer, forms the basis of the intervention. The home trainer is connected to an all-in-one computer equipped with software (Simultrainer Europe Ltd.) that provides visual feedback and virtual coaching (together with the aerobic workout, this combination is referred to as “exergaming”). Patients are instructed to train at least 3 times a week for 30–45 minutes within a predetermined heart rate zone. The heart rate zone is based on the heart rate reserve (HRR), calculated with the Karvonen method; the lower bound will gradually increase from 50% to 70% of the HRR (the upper bound is set at 80%). During the training session, the actual heart rate and time exercised within the heart rate zone, is visualized providing direct feedback. The software offers the opportunity to (a) cycle classical bike routes accompanied by real-life videos of the route, (b) cycle in a virtual world in which they can compete against their own best previous performance or virtual competitors; and (c) to perform an adjusted arcade game that is driven by the patient’s cycling performance. Taken together, this serves to motivate the patients *during* the actual exercise.

In addition, we designed a customized tablet-based motivational app (Park-in-Shape app, designed by IJsfontein Interactive Media Ltd.) to motivate patients both *before* and *after* the actual exercise. Specifically, the app shows exercise goals, provides support, and gives feedback about performance. Patients can view their results in a comprehensible way and can invite supporters to track their progress. Supporters can stimulate them to reach their goals by cheering them on, complimenting them on their performance, and promising incentives when they reach one of their goals.

Every fortnight a coach will have telephone contact with the participants to check their progress, to adjust their training schedule (if necessary) and to provide support. At least every 4 weeks the training schedules are evaluated and if the patient was easily able to adhere to the prescribed heart rate zone, the heart rate zone is raised. This allows for a graded increase in the aerobic exercise and offers a reward system by setting goals. The coach can decide on the individual intervals of these fitness checks. Communication between the bike computer and the motivational app ensures that adjustments in exercise goals are immediately and correctly enforced. Data from the training sessions are automatically saved after each session and uploaded to a secured server, allowing the coach to track the progress from a distance.

#### Stretching (active control)

The control condition is designed as an ‘active control’ to increase compliance, and consists of stretching, flexibility and relaxation exercises. The exercises do not have an aerobic component and are based on previous studies in which stretching served as a control [52,53]. The core activities encompass stretches involving the upper body and lower extremities, with the use of gentle joint extension and flexion and trunk rotation. In addition abdominal breathing with an emphasis on inhaling and exhaling to maximum capacity and relaxation of major muscles will be used. Patients are instructed to do the exercises 3 times a week for approximately 30 minutes without adding other elements to the training (no dumbbells, etcetera). After randomization, the app and the first exercise program will be explained by the coach during a home visit. The rest of the time the exercises will be executed unsupervised within the patients’ home. These exercises are commonly given to patients by the patients’ physiotherapists without specific supervision, and the majority of the exercises will be performed in seated or supine position reducing the fall risk.

In order to increase comparability between the two groups, the active control group will also be supported and motivated by the coach using the same protocol as in the intervention group. This includes an adjusted version of the motivational app for the active control group. Exercises are explained in the app through text and videos, and every month a different set of exercises will be presented. Patients will note the frequency of the performed exercises in the app, allowing the coach to follow the performance of the patients on a distance. Moreover the coach can tailor the program to the individual needs.

#### Drop-outs and adverse events

Patients who drop out will be encouraged to complete the follow-up measurements. All adverse events will be noted in the electronic database; in case of serious adverse events the responsible authorities will be notified immediately.

#### Medication adjustments

During participation, no adjustments will be made in the Parkinson medication by the research team, and participants are asked to keep their medication stable during the intervention period. However, if medication changes are deemed necessary by the treating neurologist, they are allowed to do so. Any changes in medication will be noted in the patient’s case record form.

### Power and sample size estimate

Our study is powered to show an effect of moderately-high intensive aerobic exercise on the MDS-UPDRS motor score after 6 months. For this, data on the expected effect size of the intervention (change in UPDRS motor score over 6 months with moderately intensive exercise (and standard deviation) compared to controls) is needed. We have performed a pilot study in 21 sedentary PD patients, of whom 11 were randomized to perform moderately intensive aerobic exercise for 6 months. The rest served as controls. The control group had an increase in UPDRS motor score of approximately 6.5 whereas the exercise group increased approximately 1.5 points over 6 months. This results in a difference of 5 points between the groups. The standard deviation of the follow-up measurement was approximately 9.

A clinical important difference for the within-patient change in UPDRS motor score is described in two studies and varies between 2.5-10.8 points [22,23]. Based on a combination of distribution-based and a triple anchor-based approach a minimal effect size was established at 2.5 points change, a moderate effect size at 5.2 points and a large effect size at 10.8 points change in moderately severe PD (H&YI-III) [23]. The influence of disease severity on the clinically important difference remains unclear. However the patient population represented in the MCID studies is similar to our study population, whereas the patients in our pilot where mainly early PD patients. Therefore we assumed a minimal difference between the control and the intervention group at 6 months on the MDS-UPDRS motor score of 3.5 points for the power analysis. Based on an effect size of 3.5 points, a standard deviation of 9 and a two sided α of 0.05, a sample size of n = 105 per group will be needed to obtain a power of 80%.

However, we will perform an ANCOVA analysis in which the baseline measurement will serve as a covariate. As previously described, a correction for the correlation between the baseline and follow-up scores should be made [54]. In our pilot study the correlation (r) was 0.7, resulting in n = 53 patients per group (105 × (1-r^2^)).

Adherence rates in previous moderately-high intensive exercise studies were around 80% [6,55,56]. In our pilot study all patients completed the intervention; however 81.4% of all exercised time was performed within the prescribed intensity. Therefore an attrition rate of between 18-20% was deemed reasonable, resulting in n = 65 patients per group.

### Data collection and management

Prior to data collection, study personnel will review the standard operating procedures (SOP) protocol manual. Assessors will be trained and certified in Good Clinical Practice (GCP) and in performing the MDS-UPDRS according to the guidelines of the Movement Disorder Society. Moreover they will be trained in the other assessments by experienced raters and will rate at least two persons with PD together with an experienced rater. At each measurement interval, data will be collected on paper forms and entered into a web-based data entry portal by the research nurse. Questionnaires are sent by email to the patients and are immediately imported in the electronic database after they are completed. Stored data will be backed up daily. Authenticated investigators will have access to the dataset from any internet access point. Monitoring of the study procedures and progress of the inclusion will be performed by a member of the hospital’s research center, who is not involved in the study. After completion of the study the database will be validated and locked before data-analysis is initiated.

### Statistical analysis plan

The primary and secondary outcomes will all be analyzed with an analysis of covariance (ANCOVA). The dependent variable will be the follow up scores; group allocation, gender and treatment status will serve as fixed factors and the baseline values, age at baseline, Hoehn & Yahr stage, disease duration will serve as covariates. The analyses will be performed on an intention-treat basis. In case of any missing data, sensitivity analysis (last observation carried forward) will be used with delta-adjusting imputation [57]. It should be noted that both an improvement of symptoms in the intervention group and a worsening of symptoms in the control group are considered equally important given the neurodegenerative nature of the disease. In order to determine the optimal effect of aerobic exercise, an additional per protocol analysis will be performed including all patients who completed the program as prescribed.

Adherence to the program will be analyzed according to intention-to-treat, but adherence to the prescribed heart rate will be analyzed per-protocol as this is part of a mediation analysis. Moreover dose-effect relationship will be explored by calculating the correlation between the total exercise volume and the delta score on the different outcomes. An interim analysis will not be performed.

## Discussion

Here we present the rationale and design of the Park-in-Shape study, an RCT that aims to provide evidence for the efficacy of aerobic exercise combined with gaming (exergaming) on PD-related symptoms. Previous studies have suggested a positive effect of aerobic exercise on several motor symptoms in PD patients. However, these studies had several shortcomings, as they (a) were either non-randomized/non-controlled or single-blind RCTs, (b) had outcome measures that lacked clinical relevance, (c) had small sample sizes, (d) included other elements besides aerobic exercise, such as resistance or balance training (heterogeneous interventions), or (e) were lab-based, making it difficult to translate the results to everyday life [3,15]. In this regard, strong elements of the Park-in-Shape study include the double-blinded randomized controlled design, the MDS-UPDRS as a valid primary outcome, the large sample size and the unique combination of home-based pure aerobic exercise combined with gaming elements and motivational aspects.

The double-blind character of the Park-in-Shape study is unique and allows for an even distribution of placebo effects in both groups. Obviously, performing an exercise trial in a double blinded fashion is only possible when an active control group is included (which may dilute the contrast with the exercise arm). An advantage to having an active control is that this will likely increase compliance and minimize drop-out rates in the control arm.

The UDPRS is the most commonly used outcome measure in PD research and has been extensively validated. It directly measures clinically relevant symptoms related to the patient’s disabilities and quality of life [58]. Clinically important change rates have been determined for the motor section. It is, however directly influenced by dopaminergic treatment. Therefore, it is essential to determine the medication state in which the scale is applied. We deliberately chose to test our patients in the *off* state, for the following reasons (for a recent discussion on this topic in exercise trials see: [59]): (a) testing in the *off* state will provide the best insight in the direct effect of the intervention on the disease itself, and is as such more suitable for a phase II trial such as the present one; (b) as both medicated and unmedicated patients are eligible, testing solely in *on* state (i.e. medicated patients are *on*, but unmedicated patients are basically *off*) might skew the results, as dopaminergic medication is known to have a large effect on clinical rating scales like the UPDRS; (c) with disease progression, the therapeutic window of the dopaminergic medication narrows, resulting in frequent and increasingly unpredictable response fluctuations. The quality of the *on* state is therefore not fully predictable and this hampers the before-after comparisons within patients. By testing in the *off* state, we hope to have better comparability between the disease states before and after the intervention.

The majority of RCTs that examined the effects of aerobic exercise on PD-related symptoms included fewer than 25 patients per arm [15]. Based on our power analysis, these numbers would offer a power of only <50%. The Park-in-Shape trial is adequately powered to find a significant change in MDS-UDPRS-III score, even with an attrition rate of 18%.

The intervention of the Park-in-Shape trial will provide insight into the effects of aerobic exercise on PD-related symptoms, but will also directly reveal its feasibility for implementation into a real life environment. Exercise performed in laboratories is much more difficult to translate to daily life, and compliance is probably not comparable. Performing exercise at home lowers the barrier for patients to engage in exercise, but still requires a strong motivation and discipline among participants. Because changes in exercise behavior are notoriously difficult to accomplish [56,60,61], the intervention of the Park-in-Shape trial entails several novelties compared to previous exercise interventions, all designed specifically to motivate patients to comply with the exercise program. First, addition of virtual reality and real life videos during cycling has not been used before in PD patients to stimulate their compliance in a home-based intervention. Interestingly, such enrichment (i.e. adding complexity and difficulty) has been used in experimental animal models of PD and is considered a main parameter for driving neuroplasticity [62,63]. Moreover, enrichment and variation were considered important aspects of a good exercise program by PD patients who participated in an exercise trial [61]. This concept of combining a cognitive stimulating virtual reality environment and motor training to increase the exercise effect is currently being explored in other PD trials as well, including by our group [64]. Compliance and inclusion in that trial, however is hampered by the high-frequency visits to the laboratory for the intervention. Second, we have added aspects of support, using fortnightly coaching by a medical professional, immediate visualization of their results and progress, as well as support by the patient’s family and friends.
